# Vimentin dephosphorylation at ser-56 is regulated by type 1 protein phosphatase in smooth muscle

**DOI:** 10.1186/s12931-016-0415-7

**Published:** 2016-07-25

**Authors:** Jia Li, Ruping Wang, Dale D. Tang

**Affiliations:** Department of Molecular and Cellular Physiology, Albany Medical College, 47 New Scotland Avenue, MC-8, Albany, New York USA

## Abstract

**Background:**

The intermediate filament protein vimentin undergoes reversible phosphorylation and dephosphorylation at Ser-56, which plays an important role in regulating the contraction-relaxation cycles of smooth muscle. The protein phosphatases that mediate vimentin dephosphorylation in smooth muscle have not been previously investigated.

**Methods:**

The associations of protein phosphatase 1 (PP1) and protein phosphatase 2A (PP2A) with vimentin in mouse tracheal rings was evaluated by co-immunoprecipitation. Lentivirus-mediated shRNA against PP1 was used to assess the role of PP1 in vimentin dephosphorylation and the vimentin-associated process in smooth muscle.

**Results:**

Co-immunoprecipitation analysis showed that vimentin interacted with PP1, but barely with PP2A, in airway smooth muscle. Knockdown of PP1 by lentivirus-mediated shRNA increased the acetylcholine-induced vimentin phosphorylation and smooth muscle contraction. Because vimentin phosphorylation is able to modulate p130 Crk-associated substrate (p130CAS) and actin polymerization, we also evaluated the role of PP1 in the biological processes. Silencing of PP1 also enhanced the agonist-induced the dissociation of p130CAS from vimentin and F/G-actin ratios (an index of actin polymerization). However, PP1 knockdown did not affect c-Abl tyrosine phosphorylation, an important molecule that controls actin dynamics.

**Conclusions:**

Taken together, these findings suggest that PP1 is a key protein serine/threonine phosphatase that controls vimentin Ser-56 dephosphorylation in smooth muscle. PP1 regulates actin polymerization by modulating the dissociation of p130CAS from vimentin, but not by affecting c-Abl tyrosine kinase.

## Background

The vimentin intermediate filament network of fully differentiated smooth muscle connects with the desmosome on the cellular membrane and links to the dense bodies in the myoplasm, which enables vimentin filaments to mediate the intercellular and intracellular force transmission in smooth muscle [1–5]. Vimentin undergoes phosphorylation at Ser-56 in a variety of cells/tissues in response to changes in environment, which plays a role in regulating various cellular functions including smooth muscle contraction [4–9]. Vimentin phosphorylation at Ser-56 regulates vimentin depolymerization and the spatial reorientation of vimentin filaments, which modulates the intercellular force transmission and contraction in smooth muscle [2–5, 9–15].

Vimentin phosphorylation at Ser-56 is regulated by p21-activated kinase 1 (PAK1) in smooth muscle. Contractile stimulation of smooth muscle induces PAK1 phosphorylation at Thr-423, an indication of PAK1 activation [4, 5, 9, 16]. PAK1 knockdown inhibits vimentin phosphorylation at this residue in response to contractile activation [4, 5, 9, 16]. PAK1 is able to directly catalyze vimentin phosphorylation as evidenced by the in vitro kinase assay [9, 16]. Vimentin phosphorylation at Ser-56 may be also mediated by other kinases. For example, Cdk5 mediates vimentin Ser-56 phosphorylation during GTP-induced secretion by neutrophils [6].

Smooth muscle contraction is dependent upon actin filament polymerization. A pool of actin monomers is added onto existing actin filaments in smooth muscle in response to contractile activation [17–23]. Inhibition of actin filament polymerization by pharmacological inhibitors or molecular approaches attenuates smooth muscle contraction with little or no inhibition of myosin light chain (MLC) phosphorylation [12, 17, 24–26]. Actin filament polymerization may promote contraction by enhancing the force transmission between the contractile units and the extracellular matrix [12, 24, 26, 27], by increasing numbers of the contractile units [12, 18, 24–26], and by strengthening the cadherin complex [27, 28]. Actin dynamics in smooth muscle is regulated in part by c-Abl tyrosine kinase [21, 22, 27, 29, 30]. In addition, vimentin phosphorylation also regulates actin polymerization by affecting the interaction of p130CAS (p130 Crk-associated substrate) with vimentin [4, 5, 9, 31].

MLC phosphorylation at Ser-19 is an important aspect of the cellular mechanisms that regulate smooth muscle contraction. MLC phosphorylation at Ser-19 increases myosin ATPase activity and initiates crossbridge cycling and force generation [32, 33]. The level of MLC phosphorylation at Ser-19 is regulated by myosin light chain kinase and myosin light chain phosphatase [34, 35].

Protein phosphatase 1 (PP1) and protein phosphatase 2A (PP2A) have been implicated in smooth muscle contraction [36, 37]. PP1 and PP2A serve as catalytic subunits and interact with other regulatory subunits (cofactors) to form holoenzymes for specific substrate dephosphorylation. The best characterized protein phosphatase in smooth muscle is MLC phosphatase, which consists of PP1, a regulatory subunit (MYPT1) and a 20-KDa subunit (M20) with unknown function [38]. MLC phosphatase dephosphorylates MLC phosphorylation at Ser-19 and may be regulated during contractile activation of smooth muscle [35, 38]. PP2A in smooth muscle has been implicated in dephosphorylating several substrates including L-type Ca^2+^ channel, BK_ca_ channel, PKC, caldesmon and calponin [36]. However, the phosphatases that mediate vimentin Ser-56 dephosphorylation in smooth muscle have not been previously investigated.

The objective of this study was to assess whether PP1 and/or PP2A have a role in vimentin dephosphorylation at Ser-56 in airway smooth muscle. We used mouse tracheal rings to investigate airway smooth muscle biology because the physiological and biochemical properties of the tissue preparations are similar to human airway smooth muscle [20, 29, 39]. Our results suggest that PP1 mediates vimentin dephosphorylation at this position during contractile activation of smooth muscle.

## Methods

### Animals

All experimental protocols were approved by the Institutional Animal Care and Usage Committee (Animal Welfare Assurance Number A3099-01). C57BL/6 mice (25 ± 5 g, 8–12 weeks old) were originally purchased from Taconic Biosciences and bred in the specific pathogen free housing of Animal Research Facility, Albany Medical College. The animal housing was kept at 21–22 °C with 45–55 relative humidity. The light/dark cycle of the housing was 7 am- 7 pm for fluorescent/LED lights and 7 pm – 7 am for red lights. The numbers of cage companions were 3–8 each based on animal ages and gender. Animals were healthy and able to breed normally. They were fed with Purina Lab Diet 5P76 and had continuous access to food and water. Both male and female mice were randomized allocated to the experimental or control groups. The experimental unit represented each trachea from individual animals.

### Measurement of tracheal ring contraction

Animals were sacrificed with overdosed pentobarbital sodium (100 mg/kg). Tracheal rings (4–5 mm long) were immediately removed and placed at room temperature in physiological saline solution (PSS) containing 110 mM NaCl, 3.4 mM KCl, 2.4 mM CaCl_2_, 0.8 mM MgSO_4_, 25.8 mM NaHCO_3_, 1.2 mM KH_2_PO_4_, and 5.6 mM glucose. The solution was aerated with 95 % O_2_-5 % CO_2_ to maintain a pH of 7.4. Tracheal rings were then placed in PSS at 37 °C in a 25-ml organ bath and attached to a Grass force transducer that had been connected to a Gould recorder or a computer with A/D converter (Grass). At the beginning of each experiment, 0.5 g passive tension was applied to tracheal rings. After 60 min equilibrium they were stimulated with 80 mM KCl repeatedly until contractile responses and passive tension reached a steady state.

For lentivirus-mediated shRNA in tissues, the thin epithelium layer of tracheal rings was removed by using forceps. They were then transduced with lentivirus encoding pan PP1 shRNA (sc-43533-V, Santa Cruz Biotechnology) or viruses for control shRNA (sc-108080, Santa Cruz Biotechnology) for 4 days. Contraction in response to agonist activation was compared before and after lentivirus transduction. For biochemical analysis, tissues were frozen using liquid nitrogen and pulverized as previously described [3, 4, 21].

### Immunoblot analysis

Pulverized tissues were lysed in SDS sample buffer composed of 1.5 % dithiothreitol, 2 % SDS, 80 mM Tris–HCl (pH 6.8), 10 % glycerol, 0.01 % bromophenol blue, phosphatase inhibitors (2 mM sodium orthovanadate, 2 mM molybdate, and 2 mM sodium pyrophosphate) and protease inhibitors (2 mM benzamidine, 0.5 mM aprotinin and 1 mM phenylmethylsulfonyl fluoride). The lysates were boiled in the buffer for 5 min and separated by SDS-PAGE. Proteins were transferred to a nitrocellulose membrane. The membrane was treated with blockers for 1 h and probed with use of primary antibody followed by horseradish peroxidase-conjugated secondary antibody (Fisher Scientific). Proteins were visualized by enhanced chemiluminescence (Fisher Scientific) using the LAS 400 Fuji imaging system or GE Amersham Imager 600 system. Antibodies against PP1, PP2A, phospho-myosin light chain (Ser-19), myosin light chain, phospho-c-Abl (Tyr-412) and pan c-Abl were purchased from Santa Cruz Biotechnology. Glyceraldehyde 3-phosphate dehydrogenase (GAPDH) antibody was purchased from Fitzgerald (MA, USA). Antibodies against vimentin and p130CAS were obtained from BD Biosciences. Phospho-vimentin (Ser-56) antibody was custom made by our laboratory as previously described [4, 9, 16]. Antibody against α-actin was acquired from Sigma-Aldrich. The levels of proteins were quantified by scanning densitometry of immunoblots (Fuji Multigauge Software or GE IQTL software). The luminescent signals from all immunoblots were within the linear range [20, 21, 28, 40, 41].

### Co-immunoprecipitation analysis

Co-immunoprecipitation analysis was used to evaluate protein-protein interactions as previously described [9, 20, 22]. Briefly, tissue extracts were incubated overnight with corresponding antibodies and then incubated for 2–3 h with 125 μl of a 10 % suspension of protein A-Sepharose beads. Immunocomplexes were washed four times in buffer containing 50 mM Tris–HCl (pH 7.6), 150 mM NaCl and 0.1 % Triton X-100. The immunoprecipitates were separated by SDS-PAGE followed by transfer to nitrocellulose membranes. The membranes of immunoprecipitates were probed with corresponding antibodies.

### Analysis of p130CAS dissociation with cytoskeletal vimentin

Cytoskeletal vimentin was collected using the method as previously described [4, 9, 10, 16, 31]. Briefly, tissues were homogenized in buffer containing 1 % Nonidet P-40, 10 % glycerol, 20 mM Hepes, pH 7.6, 150 mM NaCl, 2 mM sodium orthovanadate, 2 mM molybdate, 2 mM sodium pyrophosphate and protease inhibitors (2 mM benzamidine, 0.5 mM aprotinin and 1 mM phenylmethylsulfonyl fluoride). The homogenates were immediately incubated in the same buffer at 37 °C for 30 min. The soluble (depolymerized) and insoluble (cytoskeletal) fractions were separated after centrifugation at 5,200 rpm, 4 °C for 30 min. The equal amount of cytoskeletal vimentin was separated by 10 % SDS-PAGE, and was transferred to nitrocellulose membranes. The membranes were cut into two parts; the upper part was probed with monoclonal p130CAS antibody. The lower part of the membrane was blotted with vimentin antibody. The ratio of p130CAS to vimentin was calculated after densitometrical analysis of immunoblots.

### Analysis of F-actin/G-actin ratios

The content of F-actin and G-actin in smooth muscle was measured using a method as previously described [21, 22]. Briefly, tissues were treated with F-actin stabilization buffer (50 mM PIPES, pH 6.9, 50 mM NaCl, 5 mM MgCl_2_, 5 mM EGTA, 5 % glycerol, 0.1 % Triton X-100, 0.1 % Nonidet P40, 0.1 % Tween 20, 0.1 % beta-mercapto-ethanol, 1 mM ATP, 1 μg/ml pepstatin, 1 μg/ml leupeptin, 10 μg/ml benzamidine). The supernatants of protein extracts were collected after centrifugation at 151,000 g for 60 min at 37 °C. The pellets were resuspended in ice-cold H_2_O plus 1 μM cytochalasin D and then incubated on ice for 1 h to dissociate F-actin. The resuspended pellets were gently mixed every 15 min. The supernatant of the resuspended pellets was collected after centrifugation at 16,100 g for 2 min at 4 °C. Equal volume of the first supernatant (G-actin) or second supernatant (F-actin) was subjected to immunoblot analysis using α-smooth muscle actin antibody (Sigma). The amount of F-actin and G-actin was determined by scanning densitometry.

### Statistical analysis

All statistical analysis was performed using Prism 6 software (Graphpad Software Inc., CA). Differences between pairs of groups were analyzed by Student-Newman-Keuls test or Dunn’s method. Values of n refer to the number of experiments used to obtain each value. *P <* 0.05 was considered to be significant. PS: Power and Sample Size Calculation software (Vanderbilt University) was used to determine the sample size.

## Results

### Treatment with okadaic acid induces airway smooth muscle contraction

**Fig. 1:**
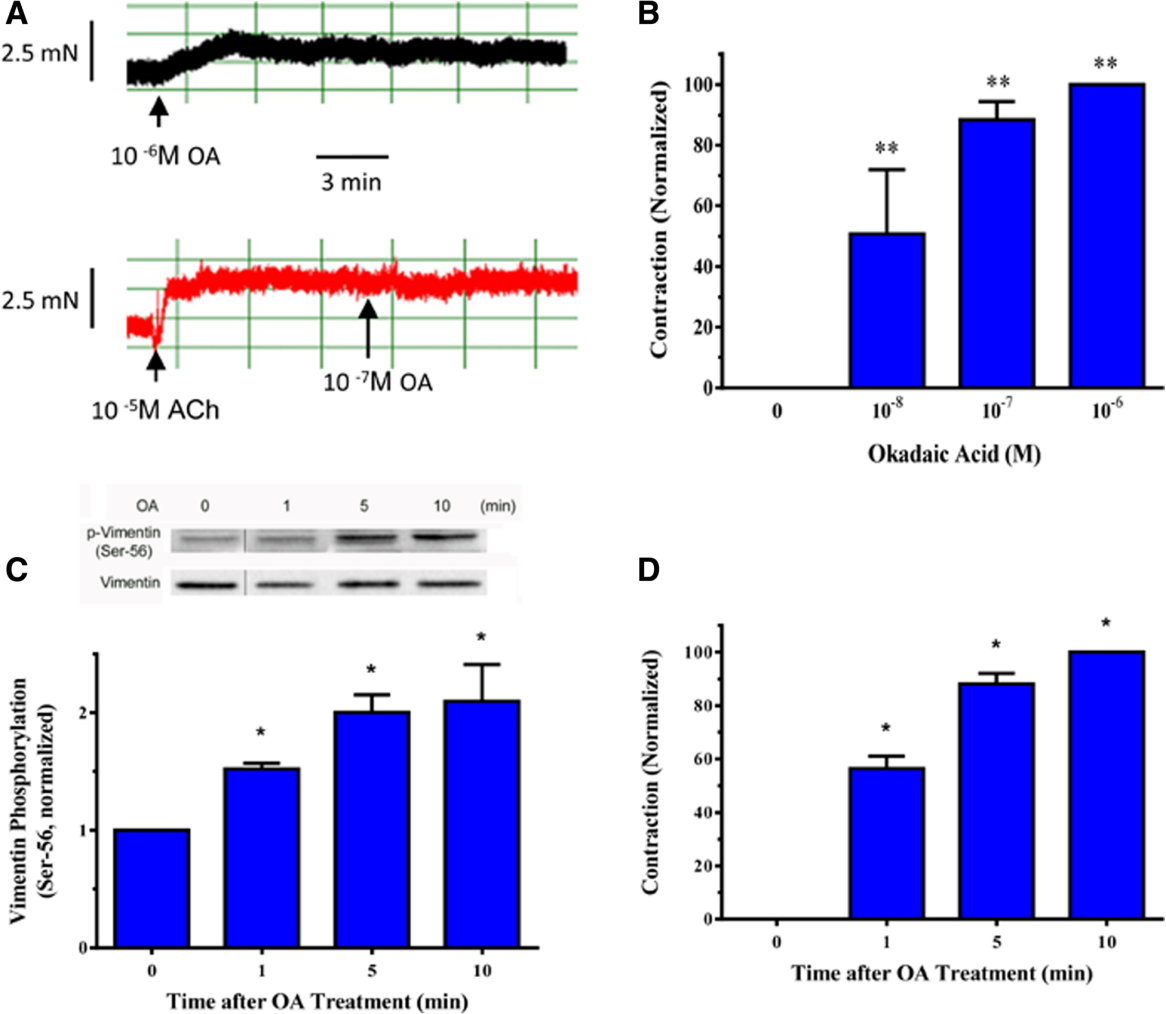
Inhibition of protein phosphatases 1 and 2A induces contraction and vimentin phosphorylation at Ser-56 in airway smooth muscle. **a** Upper panel: A representative force tracing illustrating the effects of okadaic acid (OA), a potent inhibitor of protein phosphatase 1 (PP1) and protein phosphatase 2A (PP2A) on mouse tracheal rings. Lower panel: A representative force tracing showing the effects of okadaic acid on the precontracted mouse tracheal rings. **b** Mouse tracheal rings were treated with different concentrations of okadaic acid. Contraction in these tissues was then evaluated. Tracheal contraction is normalized to the maximal contraction induced by okadaic acid. Data represent mean ± SE (*n =* 5). **, *p* < 0.01 vs. untreated tissues. **c** Mouse tracheal rings were treated with 10^−6^M okadaic acid for 1 – 10 min. Vimentin phosphorylation at Ser-56 in tissues was evaluated by immunoblot analysis. OA-induced vimentin phosphorylation is normalized to the value of untreated tissues. Vimentin phosphorylation is significantly higher in rings treated with okadaic acid than in untreated rings (*, *p* < 0.05). Data represent mean ± SE (*n =* 7–11). **d** Contraction is normalized to the maximal contraction induced by okadaic acid (10^−6^ M, 10 min). Data represent mean ± SE (*n =* 7–11). *, *p* < 0.05 vs. untreated tissues

Although protein phosphatases have been implicated in vascular and uterine smooth muscle contraction [36, 37], the role of protein phosphatases in airway smooth muscle is not well elucidated. Mouse tracheal rings were treated with okadaic acid, a potent PP1 and 2A inhibitor [36, 37]. Contractile responses were then measured using a muscle research system [4, 5, 20, 28, 29, 31]. A representative force tracing after treatment with okadaic acid was shown in Fig. 1a. Treatment with okadaic acid induced mouse tracheal smooth muscle contraction, which was dose-dependent (Fig. 1b). However, treatment with okadaic acid did not affect the ACh-induced contraction (Fig. 1a). The results suggest that the inhibition of PP1 and/or PP2A induces airway smooth muscle contraction, but not sensitize the precontracted smooth muscle.

### Treatment with okadaic acid increases vimentin phosphorylation at Ser-56 in smooth muscle tissues

Previous studies suggest that vimentin phosphorylation at Ser-56 is critical for smooth muscle contraction [3–5, 9, 12, 27]. Vimentin phosphorylation at this position regulates smooth muscle contraction by inducing the redistribution of signaling molecules and the spatial reorientation of the vimentin network [3–5, 9]. Furthermore, vimentin phosphorylation at Ser-56 is catalyzed by PAK1 [4, 5, 9, 12, 16]. However, it is currently unknown which phosphatases mediate vimentin Ser-56 dephosphorylation in smooth muscle. Mouse tracheal smooth muscle tissues were treated with okadaic acid for 1–10 min. Vimentin phosphorylation at Ser-56 and contraction were then evaluated. Treatment with okadaic acid induced vimentin phosphorylation at Ser-56 (Fig. 1c), which was coordinately associated with an increase in smooth muscle contraction (Fig. 1d). These results suggest that PP1 and/or PP2A may mediate vimentin dephosphorylation at this position in airway smooth muscle.

### Contractile activation induces the dissociation of PP1 from vimentin in smooth muscle tissues

To assess the role of PP1 and/or PP2A, we evaluated the interaction of the phosphatases with vimentin. Smooth muscle tissues were stimulated with acetylcholine (ACh), or left unstimulated. Extracts of these tissues were immunoprecipitated with vimentin antibody and immunoblotted with antibodies against vimentin, PP1 or PP2A.

**Fig. 2:**
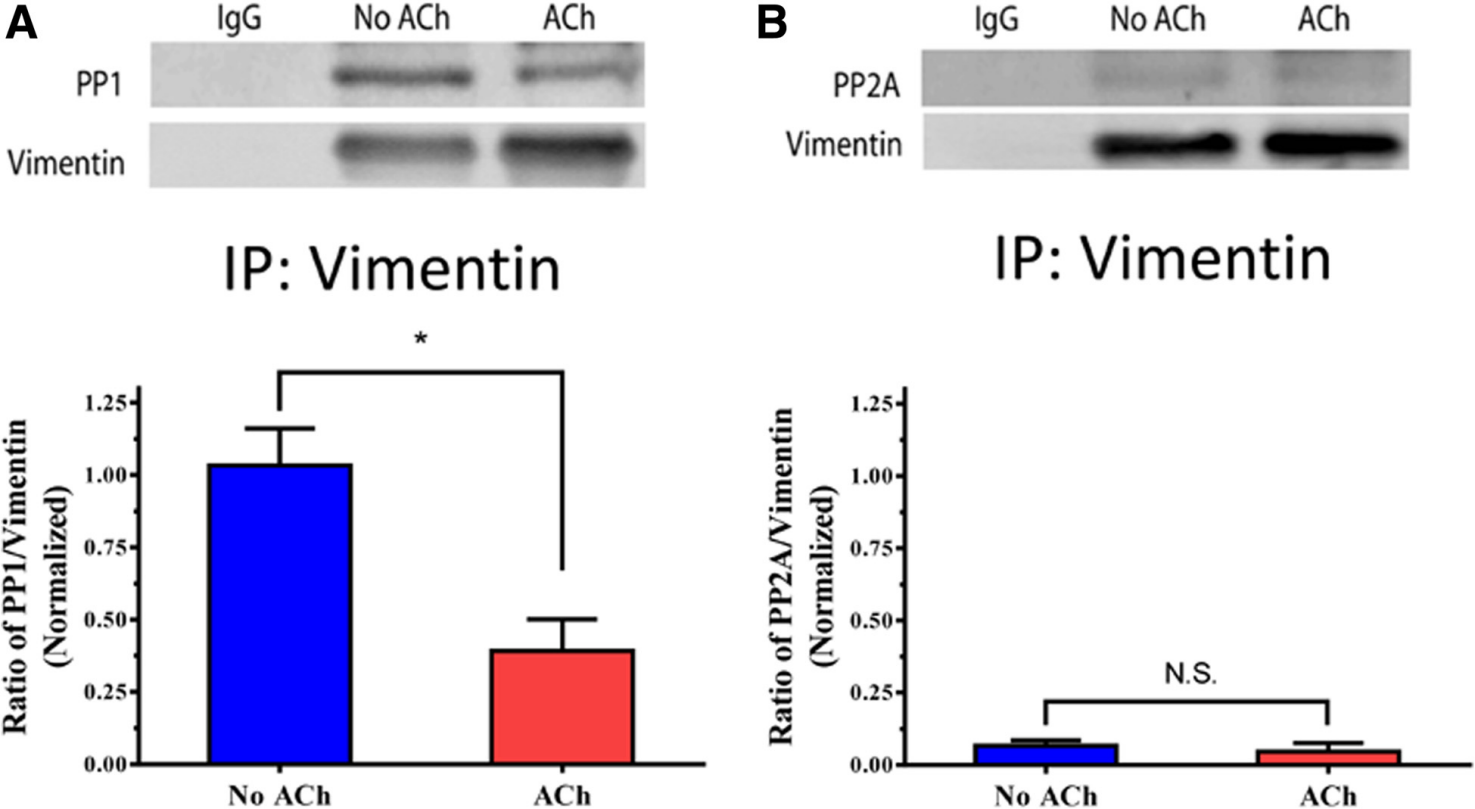
Contractile activation induces the dissociation of PP1, but not PP2A, from vimentin in smooth muscle. **a** Representative immunoblots illustrating the interaction of PP1 with vimentin. Mouse tracheal rings were stimulated with acetylcholine (ACh, 10^−4^ M, 5 min) or left unstimulated. Extracts of these tissues were immunoprecipitated using vimentin antibody, and blotted with use of antibodies against PP1 and vimentin. PP1 antibody, but not IgG, immunoprecipitates PP1 and vimentin. The ratios of PP1 to vimentin in the ACh-treated tissues are normalized to those in untreated tissues. *Significantly lower ratio of PP1/vimentin in ACh-treated rings as compared to untreated tissues (*p* < 0.05). Data are mean ± SE (*n =* 5). **b** Extracts of tissues treated with or without ACh (10^−4^ M, 5 min) were immunoprecipitated with vimentin antibody, and blotted with use of antibodies against PP2A and vimentin. PP2A is barely present in vimentin immunoprecipitates. The ratios of PP2A to vimentin in the ACh-treated tissues are normalized to those in untreated tissues. N.S., not significant (*p* < 0.05). Data are mean ± SE (*n =* 5)

In unstimulated tissues, PP1 was detected in vimentin immunoprecipitates. Upon stimulation with ACh, the ratios of PP1 over vimentin immunoprecipitates were reduced as compared to unstimulated tissues (Fig. 2a). However, PP2A was barely found in the precipitates. Moreover, the ratios of PP2A/vimentin were not significantly affected by contractile stimulation (Fig. 2b). The results suggest that vimentin predominantly interacts with PP1 in unstimulated smooth muscle. Contractile activation induces the dissociation of PP1 from vimentin in smooth muscle. We conclude that PP1 is likely a candidate for vimentin Ser-56 dephosphorylation in smooth muscle.

### Knockdown of PP1 increases vimentin phosphorylation at ser-56 and smooth muscle contraction

**Fig. 3:**
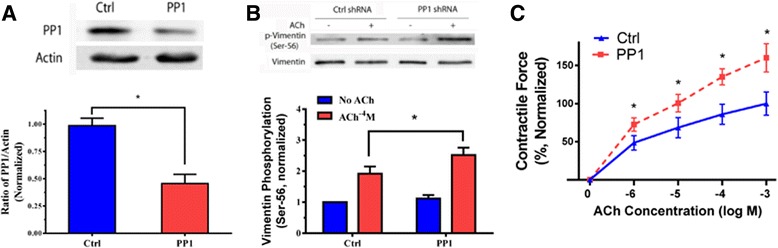
Knockdown of PP1 increases vimentin phosphorylation at Ser-56 and contraction in smooth muscle. **a** Mouse tracheal rings were transduced with lentiviruses encoding control shRNA or PP1 shRNA. These tissues were then incubated in the serum-free medium for 4 days. Immunoblot analysis was used to assess protein expression in tissues. Ctrl, control shRNA; PP1, PP1shRNA. *Significantly lower protein ratios of PP1/actin in tissues transduced with virus encoding PP1 shRNA than in tissues expressing control shRNA (*p* < 0.05). Data represent mean ± SE (*n =* 5). **b** Tracheal rings treated with control or PP1 shRNA were stimulated with ACh (10^−4^ M, 5 min), or left unstimulated. Vimentin phosphorylation at Ser-56 was evaluated by immunoblotting. Vimentin phosphorylation is normalized to unstimulated rings treated with control shRNA. Data are mean values of eight independent experiments. Error bars indicate SE (*, *p* < 0.05). **c** Contraction of tracheal rings was evaluated, after which they were transduced with lentiviruses as described above. Contractile responses were compared before and after incubation. Contraction is normalized to the contraction of rings treated with control shRNA in response to stimulation with 10^−3^ M ACh. *Significantly higher contraction of rings treated with PP1 shRNA as compared to tissues infected with viruses encoding control shRNA (*p* < 0.05). Data are mean ± SE (*n =* 6)

To determine the role of PP1, we utilized a lentivirus-mediated shRNA approach [19–21, 28, 41] to inhibit the expression of PP1. Contractile response of mouse tracheal rings to ACh was evaluated. The rings were then transduced with lentiviruses encoding control shRNA or PP1 shRNA. These tissues were incubated in the medium for 4 days. Immunoblot analysis verified the lower expression of PP1 in tissues transduced with viruses encoding PP1 shRNA compared to tissues transduced with viruses for control shRNA (Fig. 3a).

We then evaluated vimentin phosphorylation at Ser-56 in these tissues. Compared to tissues expressing control shRNA, basal vimentin phosphorylation at Ser-56 was not significantly altered in tissues expressing PP1 shRNA. More importantly, ACh-induced vimentin phosphorylation at Ser-56 was higher in PP1-deficient tissues than in tissues expressing control shRNA (Fig. 3b). We also determined the effects of PP1 knockdown on smooth muscle contraction. In tissues treated with lentivirus expressing control shRNA, ACh stimulation was able to stimulate contractile response. Moreover, contraction in PP1-deficient tissues was higher as compared to tissues expressing control shRNA (Fig. 3c). Passive tension in PP1-deficient tissues was slightly increased. We conclude that PP1 mediates vimentin dephosphorylation at Ser-56 and inhibits smooth muscle contraction.

### PP1 regulates disassociation of p130CAS from cytoskeletal vimentin in smooth muscle

**Fig. 4:**
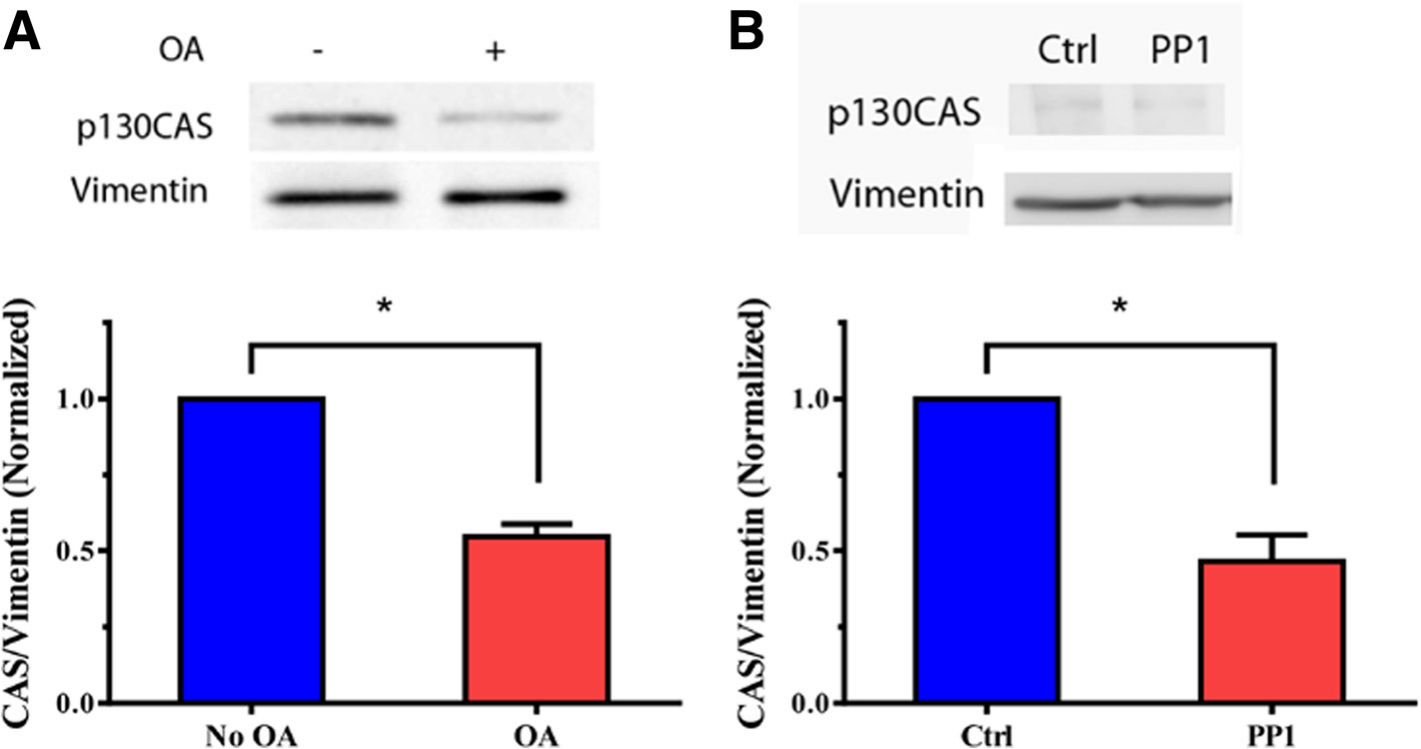
Dissociation of p130CAS from vimentin is regulated by PP1. **a** Equal amount of insoluble vimentin from untreated and okadaic acid (OA, 10^−6^ M)-treated tracheal rings were separated by SDS-PAGE, and transferred to nitrocellulose membranes. The membranes were probed with use of p130CAS antibody and vimentin antibody. The ratios of p130CAS to vimentin in the OA-treated tissues are normalized to those in untreated tissues. *Significantly lower ratio of p130CAS/vimentin in treated rings as compared to untreated tissues (*p* < 0.05). Data are mean ± SE (*n =* 5). **b** Tracheal tissues expressing control (Ctrl) or PP1 shRNA were stimulated with 10^−4^ M ACh for 5 min. The ratios of p130CAS/vimentin in tissues treated with PP1 shRNA are normalized to the ratios in tissues treated with control shRNA. Data are mean ± SE (*n =* 4). *,*p* < 0.05

Our previous studies have shown that vimentin phosphorylation enhances the disengagement of p130CAS from vimentin, which may promote smooth muscle contraction [3–5, 10, 11]. We determined the effects of okadaic acid on the association of p130CAS with vimentin. Cytoskeletal vimentin for smooth muscle tissues was collected using the intermediate filament fractionation assay [4, 5, 9, 31]. The equal amount of cytoskeletal vimentin was separated by SDS-PAGE and immunoblotted with antibodies against vimentin and p130CAS. In tissues not treated with okadaic acid, the amount of p130CAS was relatively higher. In contrast, the amount of p130CAS in cytoskeletal vimentin was reduced in rings treated with okadaic acid. The ratios of p130CAS/vimentin were reduced upon treatment with okadaic acid (Fig. 4a).

We also assessed the effects of PP1 knockdown on the interaction of p130CAS with vimentin. Compared to tissues expressing control shRNA, the amount of p130CAS in the vimentin fraction was lower in PP1-deficient tissues during ACh stimulation (Fig. 4b). Taken together, we conclude that PP1 is able to inhibit the disassociation of p130CAS from the vimentin cytoskeleton.

### Actin dynamics is regulated by PP1 in smooth muscle in response to contractile activation

**Fig. 5:**
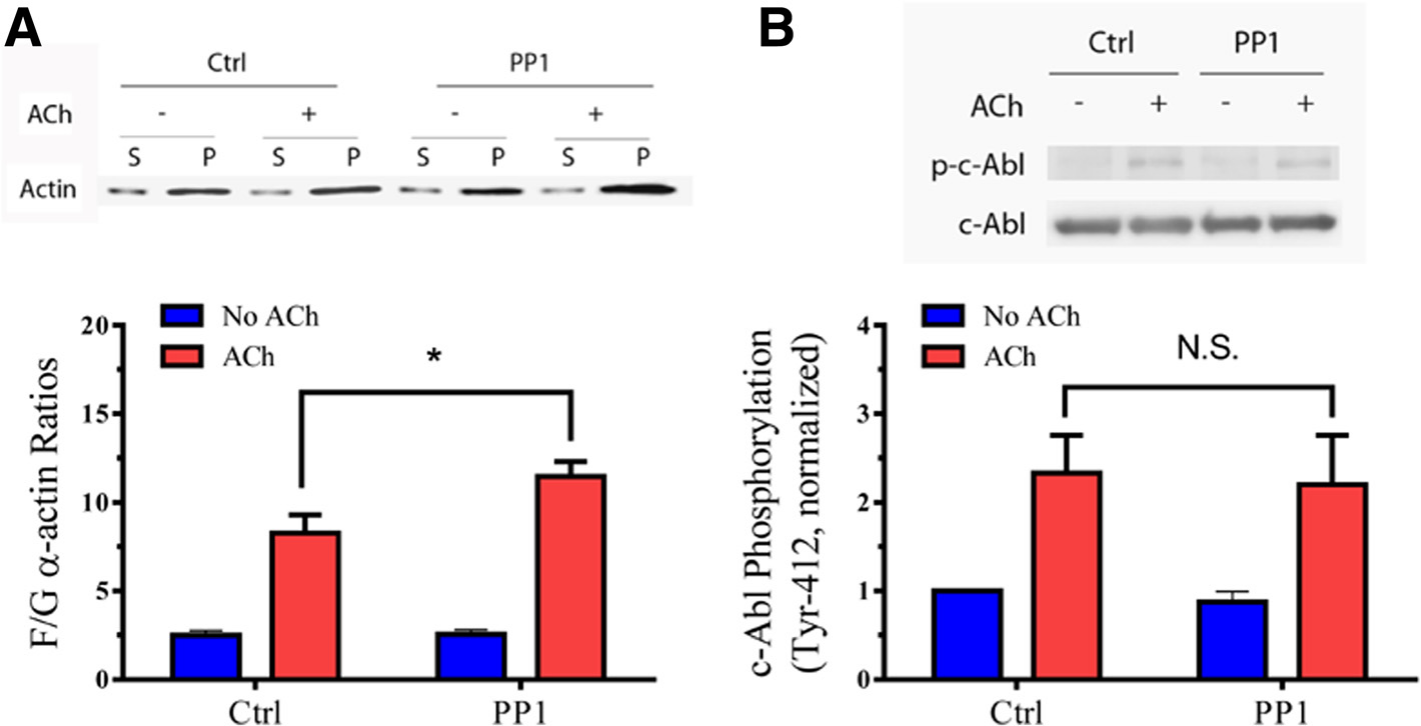
PP1 modulates ACh-induced actin polymerization, but not phosphorylation of c-Abl tyrosine kinase. **a** Tracheal rings treated with control (Ctrl) or PP1 shRNA were stimulated with 10^−4^ M ACh for 5 min, or left unstimulated. F/G-actin ratios in the tissues were evaluated using the actin fractionation assay. The ratios of F/G-actin under various treatments are normalized to the ratios in unstimulated tissues treated with control shRNA. Data are mean ± SE (*n =* 7). *, *p* < 0.05. **b** c-Abl phosphorylation at Tyr-412 in tissues treated with control or PP1 shRNA in the presence or absence of ACh was assessed by immunoblot analysis. c-Abl phosphorylation is normalized to the values in unstimulated rings treated with control shRNA. Data are mean ± SE (*n =* 4). N.S., not significant

It is well recognized that smooth muscle contraction is regulated by actin filament polymerization [12, 24, 26, 27]. Because PP1 is able to regulate smooth muscle force development, this raises a possibility that PP1 may modulate actin dynamics. To test this, we assessed the effects of PP1 knockdown on actin polymerization by using the actin fractionation assay [20–22, 28]. In tissues treated with control shRNA, ACh stimulation was able to increase F/G-actin ratios, an indication of actin polymerization [20, 24]. Furthermore, the ACh-induced increases in F/G-actin ratios were higher in PP1-deficient tissues than in tissues expressing control shRNA (Fig. 5a). The results suggest that PP1 inhibits the agonist-induced actin filament assembly in smooth muscle.

### Phosphorylation of c-Abl tyrosine kinase is not affected by PP1

c-Abl is a nonreceptor protein tyrosine kinase that regulates actin dynamics and smooth muscle contraction [21, 22, 27, 29, 30]. Since PP1 silencing affects actin filament polymerization, we questioned whether PP1 has a role in regulating c-Abl tyrosine kinase. Tissues expressing control shRNA and PP1-deficient tissues were stimulated with ACh, or left unstimulated. c-Abl phosphorylation at Tyr-412, an indication of c-Abl activation [12, 22, 24, 27], in these tissues was evaluated by immunoblot analysis. The ACh-induced c-Abl tyrosine phosphorylation in PP1-deficient tissues was similar to that in tissues expressing control shRNA. In addition, basal c-Abl phosphorylation was not affected by PP1 knockdown (Fig. 5b). We conclude that PP1 is not involved in the phosphorylation of c-Abl tyrosine kinase.

### PP1 regulates dephosphorylation of myosin light chain phosphorylation at ser-19

**Fig. 6:**
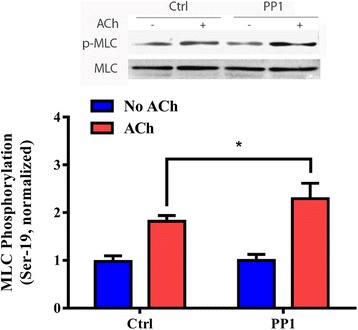
PP1 regulates myosin light chain phosphorylation at Ser-19 in smooth muscle. Mouse tracheal rings treated with control (Ctrl) or PP1 shRNA were stimulated with 10^−4^ M ACh for 5 min, or left unstimulated. Myosin light chain (MLC) phosphorylation at Ser-19 in the tissues was evaluated by immunoblot analysis. MLC phosphorylation under various treatments is normalized to the phosphorylation in unstimulated tissues treated with control shRNA. Data are mean ± SE (*n =* 4). *, *p* < 0.05

Because myosin light chain phosphorylation at Ser-19 is one of the major cellular processes in smooth muscle in response to agonist stimulation [32–35], we determined the effects of PP1 knockdown on myosin light chain phosphorylation. Compared to tissues expressing control shRNA, myosin light chain phosphorylation at Ser-19 upon ACh stimulation was higher in PP1- deficient tissues (Fig. 6). Knockdown of PP1 did not significantly affect myosin light chain phosphorylation without ACh stimulation (Fig. 6).

Since PP1 knockdown enhances the ACh-induced vimentin phosphorylation at Ser-56 (Fig. 3b), myosin light chain phosphorylation (Fig. 6) and contraction (Fig. 3c), it is likely that PP1 regulates smooth muscle contraction by controlling both the vimentin-associated pathway and myosin light chain-associated pathway.

## Discussion

The intermediate filament protein vimentin gets phosphorylated at Ser-56 in cells/tissues in response to changes in external environment, which has a role in regulating various cellular functions including smooth muscle contraction [4–9]. PAK1 has been implicated in promoting vimentin phosphorylation at Ser-56 in smooth muscle [4, 5, 9, 16]. The nature of protein serine/threonine phosphatases that mediate vimentin dephosphorylation in smooth muscle has not been previously described.

Treatment with the PP1 and PP2A inhibitors okadaic acid and calyculin A induced vimentin phosphorylation at Ser-71 in NIH3T3 cells. But, 10–20 nM calyculin A was able to induce vimentin phosphorylation at Ser-71 whereas higher doses (3–10 μM) of okadaic acid were required to induce vimentin phosphorylation. Because okadaic acid and calyculin A inhibit PP2A with similar potency, the authors concluded that PP1 is a major protein phosphatase that mediates vimentin phosphorylation at Ser-71 [42]. In contrast, treatment with okadaic acid, but not tautomysin (a putative PP1 inhibitor) induced vimentin phosphorylation in human Hs68 fibroblasts [43]. The authors concluded that PP2A, but not PP1, dephosphorylates vimentin in these cells [43]. In this report, we also observe that treatment with okadaic acid increases vimentin phosphorylation at Ser-56 in smooth muscle. Because the inhibitors are not specific, we also used lentivirus-mediated shRNA against PP1to specifically downregulates PP1 expression. PP1 knockdown enhances the agonist-induced vimentin Ser-56 phosphorylation. Our results suggest that PP1 is a predominant protein serine/threonine phosphatase that mediates vimentin dephosphorylation at Ser-56 in smooth muscle. To the best of our knowledge, this is the first evidence to suggest that PP1 controls vimentin dephosphorylation in smooth muscle. However, we do not rule out the possibility that PP2A may mediate vimentin dephosphorylation at other residues or in nonmuscle cell types.

Relatively few protein serine/threonine phosphatases regulate the dephosphorylation of thousands of phosphoprotein substrates. Typically, protein phosphatases such as PP1 and PP2A serve as catalytic subunits and interact with other cofactors to achieve specific substrate dephosphorylation. In smooth muscle, MLC phosphatase is comprised of PP1, MYPT1 and a 20-KDa subunit (M20) with unknown function. MYPT1 is able to interact with myosin light chain and renders MLC phosphatase specific. In addition, MYPT1 also regulates the functional state of MLC phosphatase [38]. We believe that the potential vimentin phosphatase (holoenzyme) should consist of PP1 and a regulatory subunit (a cofactor) at least. Future studies are required to identify the potential regulatory subunit of vimentin phosphatase.

In smooth muscle, the activity of MLC phosphatase is regulated by MYPT1 phosphorylation at Thr-696 and/or Thr-853 in vascular, gastrointestinal and myometrial smooth muscle [36]. Phosphorylation of these residues may inhibit the activity of MLC phosphatase, which increases MLC phosphorylation at Ser-19 and smooth muscle contraction [35, 36]. In this study, contractile activation induces the dissociation of PP1 with vimentin. Furthermore, agonist stimulation also results in an increase in vimentin phosphorylation at Ser-56 and contraction. The results indicate that PP1 activity may be regulated in smooth muscle in response to contractile activation. It is likely that contractile activation may inhibit PP1 activity, and/or results in the dissociation of PP1 from vimentin, which enhances vimentin phosphorylation at Ser-56 and smooth muscle contraction.

Both vimentin phosphorylation at Ser-56 and actin polymerization participate in the regulation of smooth muscle contraction. There is crosstalk between the vimentin network and the actin cytoskeleton [5, 12, 27]. Vimentin phosphorylation at Ser-56 is capable of regulating the interaction of p130CAS with the vimentin cytoskeleton. p130CAS is an adapter protein that promotes actin polymerization by activating the N-WASP/Arp2/3 pathway [4, 5, 22, 26, 44]. In unstimulated smooth muscle, approximately 50 % of total p130CAS interacts with cytoskeletal vimentin, which hinders the p130CAS-mediated actin polymerization. Upon contractile stimulation, vimentin undergoes phosphorylation at Ser-56, which releases p130CAS from the vimentin cytoskeleton and provides more “free” p130CAS for actin polymerization [4, 5, 9, 31]. In this study, knockdown of PP1 increases the dissociation of p130CAS from cytoskeletal vimentin and actin polymerization. Because actin dynamics in smooth muscle is regulated in part by c-Abl tyrosine kinase [21, 22, 27, 29, 30], we also assessed the effects of PP1 silencing on c-Abl phosphorylation. PP1 has no role in regulating c-Abl tyrosine phosphorylation in smooth muscle upon contractile stimulation. These results are consistent with the notion that PP1 regulates vimentin dephosphorylation at Ser-56, which in turn controls the association of p130CAS with cytoskeletal vimentin and actin filament assembly in smooth muscle during contractile activation.

**Fig. 7:**
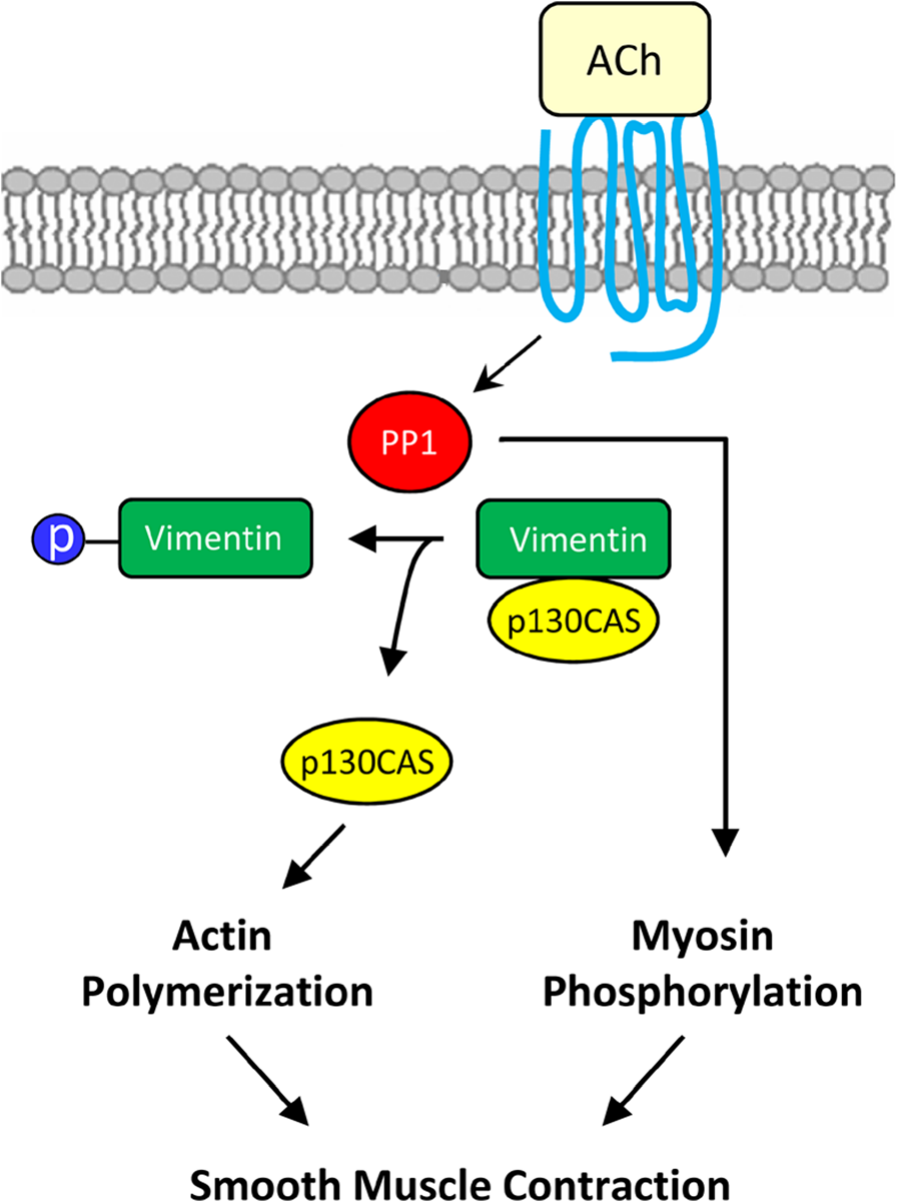
Proposed regulation of vimentin by protein phosphatase 1(PP1). Upon agonist simulation, PP1 is dissociated from vimentin and inhibited, which increases vimentin phosphorylation at Ser-56. Phosphorylated vimentin promotes the dissociation of p130CAS from cytoskeletal vimentin, which provides more “free” p130CAS for actin polymerization. In addition, PP1 may regulate myosin light chain phosphorylation. Both actin dynamics and myosin activation are indispensable for smooth muscle contraction

PP1 and PP2A have been proposed to regulate smooth muscle contraction by dephosphorylating contraction-associated proteins [36, 37]. In smooth muscle, PP1 has been proposed to regulate the dephosphorylation of myosin light chain, K^+^ channels and L-type Ca^2+^ channel whereas PP2A has been implicated in dephosphorylating several substrates including PKC, caldesmon and calponin [36]. Our present results suggest that PP1 is able to mediate vimentin dephosphorylation at Ser-56 in addition to MLC dephosphorylation at Ser-19. Thus, we propose that PP1 activity in smooth muscle is attenuated in response to contractile stimulation, which inhibits the dephosphorylation of phospho-vimentin (Ser-56) and phospho-MLC (at Ser-19). Vimentin phosphorylation may promote smooth muscle contraction by increasing intercellular force transmission [3, 5, 8, 9, 12] and p130CAS-mediated actin polymerization [4, 5, 26, 27, 31, 45]. Myosin phosphorylation may initiate crossbridge cycling and myofilament sliding. Both vimentin phosphorylation and myosin phosphorylation are needed for smooth muscle contraction (Fig. 7).

## Conclusions

Vimentin gets phosphorylated at Ser-56 in response to contractile activation, which regulates smooth muscle contraction. PAK1 has been shown to regulate vimentin Ser-56 phosphorylation in smooth muscle. The protein phosphatase that controls vimentin dephosphorylation in smooth muscle has not been previously investigated. Our present study reveals a new and novel role of PP1 in regulating vimentin dephosphorylation at this position during contractile activation of smooth muscle, which may regulate the interaction of p130CAS with cytoskeletal vimentin, actin polymerization and smooth muscle contraction (Fig. 7).

## Abbreviations

ACh, acetylcholine; Arp, actin-related protein; BKchannels, big potassium channels; GAPDH, glyceraldehyde 3-phosphate dehydrogenase; MLC, myosin light chain; MYPT1, myosin phosphatase target subunit 1; N-WASP, neuronal Wiskott-Aldrich syndrome protein; OA, okadaic acid; p130 CAS, p130 Crk-associated substrate; PAK1, p21-activated kinase 1; PKC, protein kinase C; PP1, protein phosphatase 1; PP2A, protein phosphatase 2A; shRNA, short hairpin RNA
